# Spatiotemporal prediction of sneeze pollutants in enclosed spaces: a CNN-LSTM approach validated by field measurements and CFD-Robotics arm twin system

**DOI:** 10.3389/frobt.2026.1846010

**Published:** 2026-05-26

**Authors:** Mingyu Zhao, Shujia Zhang, Ke Li, Yanru Zhang, Xiyi Wang, Tianyue Chen, Yawen Ren, Yuxi Lu, Jiahe Wang

**Affiliations:** 1 Department of Architecture, Taiyuan University of Technology, Taiyuan, China; 2 College of Architecture and Design, Shanxi Vocational University of Engineering Science and Technology, Jinzhong, China; 3 Institute of Coal Chemistry, Chinese Academy of Sciences, Taiyuan, China; 4 Shanghai Research Institute for Intelligent Autonomous Systems, Tongji University, Shanghai, China

**Keywords:** airborne transmission, computational fluid dynamics, deep learning, dynamic safety radius, robotics twin system, sneeze pollutants, spatiotemporal prediction

## Abstract

Densely populated enclosed environments, characterized by complex thermal stratification and overlapping breathing zones, represent high-risk clusters for the airborne transmission of respiratory pathogens. To address the dual challenges of physical boundary fidelity and prohibitive computational latency in traditional solvers, this study proposes a physics-validated, data-driven framework for the rapid spatiotemporal prediction of sneeze-induced pollutant dispersion. A CFD-Robotics Twin system was developed, utilizing an anthropomorphic manipulator governed by a Transformer-based Imitation Learning policy to replicate non-linear human sneezing kinematics. To ensure rigorous physical fidelity, the simulated flow fields and particle trajectories were validated through a dual-stage experimental benchmark involving anemometer measurements and robotic-arm-nozzle discharge tests. On this basis, a spatial-preserving U-ConvLSTM architecture was developed. By integrating a convolutional U-Net encoder with ConvLSTM layers and symmetric skip-connections, the model maintains absolute physical coordinates (x,y) and bypasses the topological destruction inherent in conventional flattened architectures. Evaluation via mass error $\left(\varepsilon_{\text{mass}}\right)$ and Center of Mass distance confirms strict adherence to Eulerian conservation laws. Results demonstrate that the surrogate model achieves a computational acceleration of three orders of magnitude while maintaining high structural similarity (Structural Similarity Index Measure = 0.992). Furthermore, the framework translates abstract concentration fields into practical engineering metrics, including the Dynamic Safety Radius and vertical exposure windows. This research provides a scientifically rigorous tool for real-time risk assessment and the optimization of indoor spatial layouts to mitigate pathogen exposure.

## 1 Introduction

The airborne transmission of respiratory infectious diseases, exemplified by the COVID-19 pandemic and seasonal influenza, poses a persistent and severe challenge to global public health security (Morawska and Milton, 2020). Among various expiratory activities, sneezing is identified as atransient, high-momentum multiphase flow event characterized by high ejection velocities and substantial pathogenic payloads (Duguid, 1946). Contrary to the traditional ballistic trajectory theory, seminal studies by Bourouiba et al. (Bourouiba et al., 2014) and Mittal et al. (Bhagat et al., 2020) revealed that a sneeze evolves into a multiphase turbulent buoyant cloud. This coherent structure traps polydisperse droplets ranging from micrometers to millimeters (Xie et al., 2007), protecting them from rapid evaporation and gravitational settling, thereby enabling pathogen-laden aerosols to remain suspended for extended periods. This phenomenon was further confirmed by Jadidi et al. (Jadidi et al., 2024), who reported that cough-generated droplet nuclei can persist in indoor environments for over 70 min. Such propagation mechanisms become critically hazardous in densely populated enclosed spaces, particularly university dormitories. Unlike typical flat-layout rooms, dormitories feature unique multi-level spatial configurations, which create distinct and overlapping breathing zones (Li et al., 2021). In such environments, the dispersion of bio-aerosols is not merely a horizontal diffusion process. Instead, it is governed by a highly complex interplay between human-induced thermal stratification and the downward gravitational settling of heavier droplets (Mittal et al., 2020). Furthermore, the inherent structural complexity of such spaces often necessitates uncertainty-aware hybrid modeling to achieve accurate spatial forecasting when structural or thermal information is incomplete (Kang et al., 2026). Consequently, quantitatively characterizing the spatiotemporal propagation laws of sneeze-induced pollutants in these specific confined settings is a prerequisite for effective risk assessment and mitigation.

To quantitatively characterize these complex dispersion processes, Computational Fluid Dynamics (CFD) has become an indispensable tool for investigating indoor airborne transmission. Specifically, the Eulerian–Lagrangian approach implemented via the Discrete Phase Model (DPM) in ANSYS Fluent is widely regarded as one of the most effective methods for tracking the trajectories and phase change of pathogen-laden droplets (Zhang and Chen, 2007). Successful applications have been extensively documented. Dbouk and Drikakis employed this framework to elucidate droplet transport dynamics during sneezing under varying wind conditions, while Blocken et al. (Blocken et al., 2021) extended its application to large-scale sports centers and air-conditioned rooms, demonstrating the decisive impact of ventilation patterns on reducing infection risks. Despite its ability to provide high-fidelity flow field information, traditional CFD simulations face inherent and severe bottlenecks in computational efficiency. Resolving multiscale turbulence structures and transient particle dynamics requires iterative solutions of the Navier–Stokes equations on extremely fine grids, resulting in prohibitive computational costs. As reported by Wang et al. and Shao et al. , a single transient simulation covering only a few minutes of biological dispersion can require hours or even days on high-performance computing clusters. This computationally intensive nature introduces significant latency, rendering conventional CFD unsuitable for scenarios requiring real-time risk assessment (Wei and Li, 2016) or iterative spatial layout optimization in which thousands of configurations must be evaluated rapidly (Faulkner et al., 2023).

To overcome the computational bottlenecks inherent in conventional CFD, data-driven deep learning approaches have emerged as a promising paradigm for constructing reduced-order models (ROMs) with real-time inference capability. Pioneering studies by Guo et al. (Guo et al., 2016) and Thuerey et al. (Thuerey et al., 2020) demonstrated the effectiveness of convolutional neural networks (CNNs) in extracting complex geometric features and flow structures. Extending these methods to multiphase droplet dynamics, Solanki et al. (Lashkaripour et al., 2021) applied CNNs to predict microfluidic droplet generation characteristics directly from geometric slices, validating the ability of deep learning models to capture complex multiphase flow behavior. In the context of indoor environments, Zhang et al. successfully employed encoder–decoder architectures to predict contaminant concentration distributions. Building on these foundations, Mesgarpour et al. developed an AI-assisted computational framework to rapidly predict sneeze-induced droplet dispersion in a bus, achieving a speedup of two orders of magnitude compared with large-eddy simulations. More recently, Yao et al. integrated CFD with a random forest algorithm to reconstruct the spatiotemporal distribution of cough droplets. However, sneeze-induced pollutant dispersion is a highly transient process, posing significant challenges to the temporal modeling capabilities of static or quasi-static approaches. To address this limitation, hybrid architectures combining CNNs with long short-term memory (LSTM) networks or ConvLSTM models have been proposed (Shi et al., 2015). Studies by Wang et al. and Shao et al. (Shao et al., 2026) demonstrated the superiority of these hybrid models in capturing time-dependent nonlinear dynamics. Despite these advances, several critical knowledge gaps remain. First, most existing data-driven studies rely exclusively on unverified numerical datasets, lacking rigorous physical validation of airflow structures and droplet trajectories through anthropomorphic experiments. Although advanced adaptive control strategies have been developed to achieve highly biomimetic and precise movements in robotic joints (Zhou et al., 2024), their application in synchronizing physical robotic systems with numerical fluid simulations for pollutant validation remains largely unexplored. Second, existing AI models are fundamentally inadequate for resolving the complex vertical transmission mechanisms in multi-level confined spaces. Conventional architectures often flatten spatial features through fully connected layers, thereby destroying the physical spatial topology. Consequently, they fail to implicitly learn the gravity-induced droplet settling and thermally-driven aerosol buoyancy. Third, existing models rarely translate concentration predictions into practical engineering metrics, such as dynamic safety distances, thereby limiting their applicability to spatial layout optimization.

To address these limitations, this study aims to establish a physics-validated, data-driven framework for the rapid prediction of spatiotemporal pollutant dispersion in enclosed dormitory environments. First, high-fidelity transient numerical simulations were conducted using the Eulerian-Eulerian Mixture Model in ANSYS Fluent to simulate the multiphase sneeze process. To address the lack of rigorous physical validation of airflow structures and droplet trajectories, a dual-stage experimental validation strategy was designed. This strategy involved validating the airflow field using anemometers at key locations and, critically, verifying droplet trajectory ranges using a physical–numerical twin system composed of a robotic arm and its synchronized numerical motion model. This digital twin framework, increasingly recognized for its ability to bridge physical execution and virtual simulation in complex environments (Muratore et al., 2022), ensures the reliability of the discrete phase model. Based on the validated physical model, a systematic spatiotemporal dataset was generated, encompassing multiple release locations and ventilation scenarios. Subsequently, to address the architectural limitations of conventional networks in capturing vertical dynamics, a spatial-preserving hybrid deep learning model, integrating a purely convolutional U-Net encoder and ConvLSTM networks, was developed. By eliminating spatial-flattening fully connected layers and utilizing skip-connections, this novel architecture inherently preserves the absolute physical coordinates of the flow field, enabling the model to accurately capture the nonlinear temporal evolution driven by gravitational settling and thermal stratification. Ultimately, this framework enables prediction of concentration fields with high accuracy, quantifies dynamic safety distances, and identifies critical exposure windows associated with vertical transmission between upper and lower bunks. These findings provide a robust scientific basis for optimizing indoor spatial layouts and facility configurations to minimize pathogen exposure risks in densely populated confined environments.

## 2 Theoretical framework

### 2.1 Overall research framework

**FIGURE 1:**
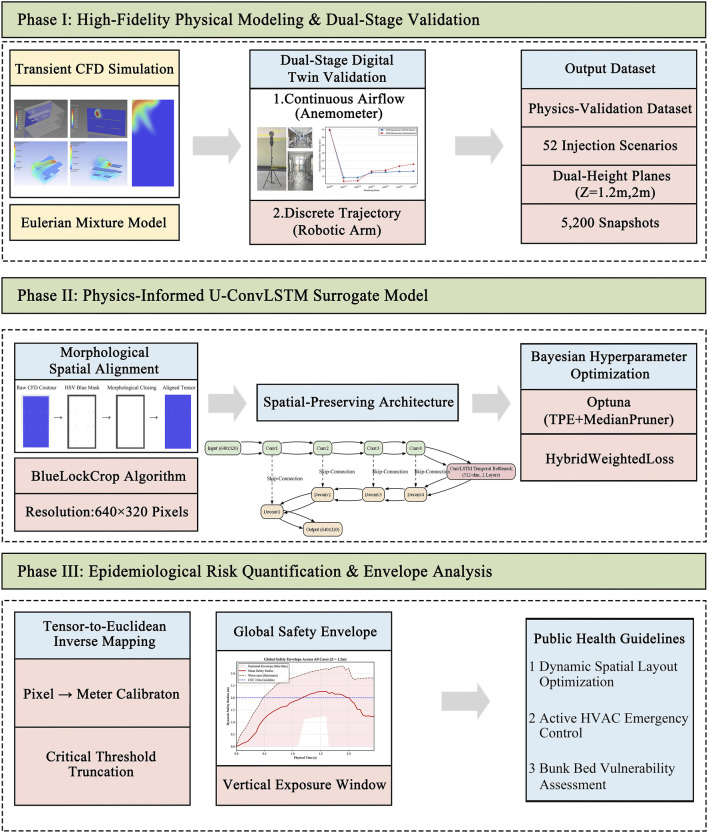
Technical route.

A physics-informed three-stage research framework was established. As illustrated in Figure 1, this framework forms a robust closed-loop spanning from rigorous physical modeling to real-time AI inference, and finally to engineering-oriented risk quantification.

The foundation of this study lies in generating a reliable aerosol dispersion database that accurately reflects the complex indoor aerodynamics. Initially, transient CFD simulations were conducted using the Eulerian-Eulerian Mixture Model to capture the multiphase fluid dynamics of sneeze events across various release locations and ventilation scenarios. Furthermore, to overcome the common vulnerability of relying solely on unverified numerical data, a dual-stage experimental system was constructed. In the first stage, an anemometer array was deployed in the physical dormitory to validate the continuous airflow fields. In the second stage, an anthropomorphic robotic arm-driven sneeze simulator was developed to physically replicate the discrete particle trajectories and maximum settling distances.

Addressing the computational bottlenecks of iterative CFD solvers and the severe spatial distortion inherent in conventional Convolutional Neural Networks (CNNs), a U-ConvLSTM architecture was proposed. The proposed purely convolutional Encoder-Decoder structure preserves the absolute 4D spatial coordinates (x,y) of the pollutant cloud via high-resolution skip-connections.

Through an automated post-processing pipeline, the normalized pixel matrices predicted by the U-ConvLSTM were mathematically mapped back to the physical dimensions of the dormitory. This crucial step enables the derivation of two vital engineering metrics: the continuous quantification of Dynamic Safety Distances, mapping the expansion and contraction of hazardous radii over time; and the identification of Critical Exposure Time Windows unveiling the hidden risks of vertical transmission between upper and lower bunks driven by thermal stratification and gravitational settling.

### 2.2 Numerical simulation of aerosol dispersion

In this study, transient computational fluid dynamics simulations were conducted using ANSYS Fluent. The computational domain represents a standard six-person university dormitory with dimensions of 6.8 m × 3.62 m × 2.8 m, characterized by a typical loft bed layout whose upper bunks are used for sleeping and lower areas for study desks. Given the objective to generate continuous concentration field data for deep learning training, the Eulerian-Eulerian Mixture Model was selected over the Lagrangian Discrete Phase Model (DPM). While DPM tracks individual particles, the Mixture model treats the multiphase system as interpenetrating continua, which is computationally more efficient for resolving the macroscopic volume fraction distributions required for image-based learning.

#### 2.2.1 Governing equations of fluid and particles

The numerical solution involves solving the continuity, momentum, and volume fraction equations for the mixture. The mixture properties, such as density and viscosity are computed as volume-fraction-weighted averages of the primary air phase and the secondary pollutant phase. The continuity equation for the mixture is expressed in Equation 1:

$$\frac{\partial }{\partial t}\left(\rho_{m}\right)+\nabla \cdot \left(\rho_{m}{\vec{v}}_{m}\right)=0$$ (1)

Where ${\vec{v}}_{m}$ represents the mass-averaged velocity. The momentum equation incorporating the sum of forces acting on all phases is defined in Equation 2:

$$\begin{array}{cc} \frac{\partial }{\partial t}\left(\rho_{m}v_{m}\right) & +\nabla \cdot \left(\rho_{m}v_{m}v_{m}\right)=-\nabla p+\nabla \cdot \left[\mu_{m}\left(\nabla v_{m}+\nabla v_{m}^{T}\right)\right]\\ & +\rho_{m}g+F-\nabla \cdot \left(\overset{n}{\underset{k=1}{\sum }}\alpha_{k}\rho_{k}v_{dr,k}v_{dr,k}\right) \end{array}$$ (2)

Furthermore, the drift velocity term models the relative motion between the dispersed phase and the mixture, capturing the sedimentation physics of pollutant droplets. The transport of these bio-aerosols is described by the volume fraction equation for the secondary phase, as shown in Equation 3:

$$\frac{\partial }{\partial t}\left(\alpha_{p}\rho_{p}\right)+\nabla \cdot \left(\alpha_{p}\rho_{p}{\vec{v}}_{m}\right)=-\nabla \cdot \left(\alpha_{p}\rho_{p}{\vec{v}}_{dr,p}\right)$$ (3)

#### 2.2.2 Boundary conditions and parameter configuration

To accurately resolve the turbulent characteristics of the indoor airflow and the high-momentum sneeze jet, the Realizable k−ε turbulence model with standard wall functions was adopted. This model provides superior performance in predicting the spreading rate of planar and round jets compared to the standard k−ε model. The material properties of the pollutant phase were defined to mimic sneeze droplets, with a density of $1050\;{\text{kg/m}}^{3}$ (Redrow et al., 2011) and a viscosity of 0.012 kg/(m⋅s) .

The boundary conditions were configured to replicate realistic physiological and environmental scenarios. The mouth of the infected individual was modeled as a velocity inlet. To simulate the transient dynamics of a violent sneeze, a User-Defined Function (UDF) was compiled to impose a time-dependent velocity profile U(t) and a droplet volume fraction profile. The sneezing event was modeled as a 0.5-s pulse with a trapezoidal velocity profile, reaching a peak velocity of U max = 15.0 m/s. The mathematical definition of this transient velocity profile is given in Equation 4:

$$U\left(t\right)=\left\{\begin{array}{cc} U_{\max}\cdot \frac{t}{0.05} & 0\leq t<0.05\;s\;\left(\text{Acceleration}\right)\\ U_{\max} & 0.05\leq t<0.45\;s\;\left(\text{Peak Hold}\right)\\ U_{\max}\cdot \left(1-\frac{t-0.45}{0.05}\right) & 0.45\leq t\leq 0.5\;s\;\left(\text{Decay}\right)\\ 0 & t>0.5\;s \end{array}\right.$$ (4)

In the Eulerian mixture model, the velocity inlet was calibrated using a discharge coefficient $\left(C_{d}\right)$ of 0.94, accounting for the internal viscous losses of the physical nozzle. To ensure hydrodynamic fidelity, the initial turbulence intensity at the inlet was defined based on the nozzle’s hydraulic diameter and the peak Reynolds number $\left(Re\approx 1.2\times 10^{4}\right)$ . This parameterization accounts for the anisotropic velocity distribution observed in experimental discharge tests, providing a more rigorous initial condition than idealized flat-velocity patches.

Simultaneously, the pollutant phase was injected during the active sneezing interval ( t≤0.5 s), and environmental boundaries included velocity inlets for windows and pressure outlets for door gaps. The simulation was executed with a transient time step of Δt=0.01 s, satisfying the Courant-Friedrichs-Lewy (CFL) condition to capture the rapid evolution of the jet.

#### 2.2.3 Contours generation and data acquisition

Data acquisition focused on the primary zones of human respiratory exposure within the dormitory. Two horizontal monitoring planes were established at characteristic heights: $Z_{\text{low}}=1.2\text{ m}$ and $Z_{\text{high}}=2.0\text{ m}$ . The 1.2 m plane corresponds to the breathing zone of occupants in a seated posture, while the 2.0 m plane corresponds to the breathing zone of occupants resting in the upper bunks. This dual-height selection is critical for evaluating the vertical transmission risks induced by thermal stratification and aerosol buoyancy.

At each time step, the instantaneous distribution of the pollutant volume fraction on these planes was extracted and mapped into 2D concentration contour matrices. To ensure that the pixel intensity I (x,y) linearly represents the physical concentration C (x,y), a global fixed range was applied to the color mapping function across all frames. This preprocessing step preserves the physical consistency of the data, enabling the neural network to learn the quantitative governing laws of dispersion.

### 2.3 Robotic kinematics and intelligent execution strategy

To rigorously validate the Eulerian-Lagrangian multiphase CFD solutions, it is imperative to establish a physical boundary condition that replicates the complex, non-linear physiological kinematics of a human sneeze (Vrigkas et al., 2015). Rather than employing rigid, stationary nozzles which fundamentally misrepresent the dynamic ejection vector, this study constructed an anthropomorphic manipulator governed by a Transformer-based Imitation Learning policy (Wolf et al., 2025).

#### 2.3.1 Anthropomorphic kinematic parameterization

The spatial configuration of the manipulator is parameterized by its joint space vector $q\left(t\right)={\left[q_{1}\left(t\right),q_{2}\left(t\right),\ldots ,q_{6}\left(t\right)\right]}^{T}\in R^{6}$ . The transient position and orientation of the end-effector are mapped to the Cartesian task space via the forward kinematics function $T_{ee}\in SE\left(3\right)$ shown in Equation 5:

$$T_{ee}\left(t\right)=\overset{6}{\underset{i=1}{\prod }}A_{i}\left(q_{i}\left(t\right)\right)$$ (5)

${\text{where}}^{i-1}A_{i}$ denotes the homogeneous transformation matrix between adjacent links derived from Denavit-Hartenberg parameters.

The anthropomorphic replication was executed by a 6-DOF SO-ARM101 manipulator. The kinematic chain, defined by the Denavit-Hartenberg (D-H) parameters in Table 1, ensures that the forward flexion trajectory matches the physiological range of a human torso. Furthermore, the R550 chassis provided a high-stability platform; its 35 kg unladen mass and low center of gravity successfully suppressed the reaction force vibrations generated by the 15 m/s fluidic pulse.

**TABLE 1:** Calibrated D-H parameters for the SO-ARM101.

Link (i)	$d_{i}$ (offset/mm)	$a_{i}$ (length/mm)	$\alpha_{i}$ (twist/rad)
1	65	0	π/2
2	0	140	0
3	0	140	0
4	0	0	π/2
5	50 (wrist)	0	−π/2
6	40 (End)	0	0

During the teleoperation phase, the physiological sneeze trajectories were manually executed on a kinematically isomorphic leader arm. The discrete temporal evolution of the joint positions and velocities was synchronously sampled to construct the expert demonstration dataset $D=\left\{\tau_{1},\tau_{2},\ldots ,\tau_{N}\right\}$ , where each trajectory $\tau_{n}=\left\{\left(q_{0},{\dot{q}}_{0}\right),\ldots ,\left(q_{T},{\dot{q}}_{T}\right)\right\}$ .

#### 2.3.2 Action chunking with transformers

To ensure the high-fidelity replication of the high-momentum expiratory action, the Action Chunking with Transformers (ACT) algorithm was theoretically implemented. Instead of predicting a singular instantaneous action, the ACT policy predicts a continuous chunk of future joint targets $A_{t}=\left[a_{t},a_{t+1},\ldots ,a_{t+k-1}\right]$ , thereby strictly preserving the temporal smoothness of the aerodynamic boundary condition.

The ACT architecture is formulated as a Conditional Variational Autoencoder (CVAE). During the training phase, the encoder $E_{\phi }$ compresses the ground-truth action sequence into a latent macroscopic stylistic variable z∼N(μ,Σ) . Subsequently, a Transformer-based decoder $D_{\theta }$ reconstructs the kinematic action chunk conditioned on both the latent variable z and the current proprioceptive state observation $s_{t}=\left(q_{t},{\dot{q}}_{t}\right)$ as defined in Equation 6:

$${\hat{A}}_{t}=D_{\theta }\left(s_{t},z\right)$$ (6)

The self-attention mechanism within the Transformer decoder inherently models the global temporal dependencies of the physiological motion, ensuring that the explosive downward flexion is flawlessly synchronized with the fluidic pulse triggering sequence.

To replicate the high-momentum transient kinetics of a human sneeze, the SO-ARM101 manipulator was operated under a high-frequency control loop of 100 Hz, ensuring millisecond-level responsiveness to the Transformer-based policy. To mitigate communication latency between the master control node and the R550 mobile chassis, a hardware-level deterministic trigger was implemented. Specifically, the fluidic discharge was synchronized with the peak angular velocity of the cervical flexion joint. By utilizing the ROS-based low-latency communication protocol inherent in the R550 chassis, the pollutant ejection vector is perfectly aligned with the mechanical maximum-acceleration point, faithfully replicating the synergistic physiological action of a violent expiratory event.

#### 2.3.3 Policy optimization objective

To optimize the neural parameters of the follower arm, the learning objective minimizes the discrepancy between the synthesized action chunks and the expert demonstration trajectories, while concurrently regularizing the latent space to prevent deterministic overfitting. The systematic loss function $\left(L_{\mathrm{ACT}}\right)$ is defined in Equation 7:

$$L_{\mathrm{ACT}}=\lambda_{1}\overset{k-1}{\underset{j=0}{\sum }}\|{\hat{a}}_{t+j}-a_{t+j}{\|}_{1}+\lambda_{2}\cdot D_{KL}\left(N\left(\mu ,\Sigma \right)\|N\left(0,I\right)\right)$$ (7)

The first term calculates the L1-norm reconstruction error, enforcing rigid kinematic fidelity to guarantee that the ejection vector of the sneeze simulator perfectly aligns with the CFD inlet condition. The second term, governed by the Kullback-Leibler (KL) divergence $\left(D_{KL}\right)$ , penalizes the deviation of the latent distribution from an isotropic Gaussian prior N(0,I) .

### 2.4 Deep learning network architecture

To accurately capture the spatiotemporal evolution characteristics of pollutant dispersion, this study constructs a hybrid deep learning model. This architecture integrates Convolutional Neural Networks (CNNs) (Srinivas et al., 2016) for spatial feature extraction and Long Short-Term Memory (LSTM) networks for temporal sequence modeling.

#### 2.4.1 Spatial feature extraction: convolutional neural networks

Convolutional Neural Networks are designed to automatically and adaptively learn spatial hierarchies of features from low-to high-level patterns. A typical CNN architecture consists of convolutional layers, pooling layers, and activation functions. The Convolutional Layer is the core building block, where a set of learnable filters convolve across the input image to extract local features such as concentration gradients and dispersion boundaries. Mathematically, the feature map value F (i,j) at position (i,j) is calculated using Equation 8:

$$F\left(i,j\right)=\left(X*K\right)\left(i,j\right)=\underset{m}{\sum }\underset{n}{\sum }X\left(i+m,j+n\right)\cdot K\left(m,n\right)+b$$ (8)

Where X represents the input matrix, K denotes the kernel, and b is the bias term. Following the convolution operation, a pooling layer is typically applied to downsample the spatial dimensions, thereby reducing computational complexity and controlling overfitting while preserving dominant features.

In traditional deep learning applications, CNNs often function as spatial encoders to transform high-dimensional inputs into compact feature vectors. As baselines in this study, three representative backbones were investigated. Common backbones such as VGG16 (Simonyan and Zisserman, 2014), ResNet (Shafiq and Gu, 2022), and EfficientNet (Tan and Le, 2019) typically employ Global Average Pooling and fully connected layers. While highly effective for classification tasks, these operations irrevocably discard the absolute spatial coordinates (x,y) of the input scalar field. In the context of transient CFD prediction, this topological destruction consistently leads to spatial distortion.

To rigorously overcome this issue, this study implements a fully convolutional encoder-decoder structure inspired by the U-Net architecture. The proposed encoder relies exclusively on strided convolutions, ensuring that the feature representations strictly maintain a 4D tensor structure (B,C,H,W) . During the decoding phase, symmetrical skip-connections are utilized. This mechanism directly concatenates the low-level, high-resolution feature maps $F_{\mathrm{enc}}^{\left(l\right)}$ from the encoder with the corresponding up-sampled feature maps $F_{\mathrm{dec}}^{\left(l\right)}$ in the decoder. This architecture intrinsically preserves the physical spatial topology of the pollutant cloud, empowering the network to reconstruct highly non-linear, sharp concentration boundaries without spatial decay.

#### 2.4.2 Temporal evolution modeling: long short-term memory

While CNNs excel at spatial interpretation, they are limited in processing time-series data. Traditional Recurrent Neural Networks (RNNs) suffer from gradient explosion or vanishing when learning long-term dependencies. To address this, Long Short-Term Memory (LSTM) networks were introduced. The core innovation of LSTM is its cell state $\left(C_{t}\right)$ , regulated by three gate structures—the Forget Gate $\left(f_{t}\right)$ , Input Gate $\left(i_{t}\right)$ , and Output Gate $\left(o_{t}\right)$ —which selectively remove or add information. This mechanism enables the model to effectively capture both the transient fluctuations and long-term diffusion trends of pollutants.

The mathematical transition equations for an LSTM unit at time step t are defined in Equation 9:

$$\begin{array}{cc} f_{t} & =\sigma \left(W_{f}\cdot \left[h_{t-1},x_{t}\right]+b_{f}\right)\\ i_{t} & =\sigma \left(W_{i}\cdot \left[h_{t-1},x_{t}\right]+b_{i}\right)\\ {\tilde{C}}_{t} & =\tanh\left(W_{c}\cdot \left[h_{t-1},x_{t}\right]+b_{c}\right)\\ C_{t} & =f_{t}⊙C_{t-1}+i_{t}⊙{\tilde{C}}_{t}\\ o_{t} & =\sigma \left(W_{o}\cdot \left[h_{t-1},x_{t}\right]+b_{o}\right)\\ h_{t} & =o_{t}⊙\tanh\left(C_{t}\right) \end{array}$$ (9)

Where σ is the sigmoid function, ⊙ denotes element-wise multiplication, and $h_{t}$ is the hidden state passed to the next step.

#### 2.4.3 Loss function for highly sparse domains

A predominant challenge in training neural networks on high-resolution indoor CFD fields is extreme spatial sparsity. In practical sneeze dispersion scenarios, over 95% of the computational domain corresponds to unpolluted ambient air, where the mass fraction C(x,y)≈0 . Relying strictly on conventional Mean Squared Error (MSE) universally biases the gradient descent towards optimizing the zero-valued background. Consequently, the network mathematically favors mean color collapse, yielding predictions where the critical high-concentration aerosol core is heavily attenuated or entirely eradicated.

To enforce physical and kinetic fidelity, this study mathematically formulated a customized HybridWeightedLoss $\left(L_{\text{total}}\right)$ . The primary component is an adaptive weighted penalty, where the weight factor Ω(x,y) scales linearly with the ground-truth concentration intensity $Y_{\text{true}}$ as expressed in Equation 10:

$$\Omega \left(x,y\right)=1.0+\left(\lambda_{\text{cloud}}-1.0\right)\cdot \max_{c\in \left\{R,G\right\}}Y_{\text{true}}^{\left(c\right)}\left(x,y\right)$$ (10)

Furthermore, a scalar peak conservation term $\left(L_{\text{peak}}\right)$ was introduced, as defined in Equation 11, to guarantee that the absolute maximum predicted pathogenic payload matches the ground-truth exposure:

$$L_{\text{peak}}=\lambda_{\text{peak}}\cdot \left|\max\left(Y_{\text{pred}}\right)-\max\left(Y_{\text{true}}\right)\right|$$ (11)

Ultimately, $L_{\text{total}}$ couples the weighted MSE, L1 loss, peak conservation loss, and Structural Similarity Index (SSIM) loss, simultaneously enforcing numerical precision and macroscopic structural topology.

### 2.5 Performance evaluation metrics

Evaluating deep learning surrogates in fluid dynamics necessitates a departure from standard computer vision benchmarks. A rigorous multi-dimensional metric framework was thus established, coupling conventional statistical error quantification with physics-informed constraints, to systematically validate the hydrodynamic fidelity and epidemiological applicability of the predicted aerosol dispersion fields.

#### 2.5.1 Global numerical accuracy: RMSE

To quantify the absolute pixel-wise numerical deviation across the spatial domain, the Root Mean Square Error (RMSE) was adopted as the primary global baseline metric. Let $Y_{\text{pred}}$ denote the spatial concentration matrix predicted by the U-ConvLSTM and $Y_{\text{true}}$ denote the corresponding CFD-derived ground truth. The RMSE is mathematically defined in Equation 12:

$$RMSE=\sqrt{\frac{1}{N\cdot H\cdot W}\overset{N}{\underset{n=1}{\sum }}\overset{H}{\underset{i=1}{\sum }}\overset{W}{\underset{j=1}{\sum }}{\left(Y_{\mathrm{pred}}^{\left(n\right)}\left(i,j\right)-Y_{\mathrm{true}}^{\left(n\right)}\left(i,j\right)\right)}^{2}}$$ (12)

Where N denotes the total number of samples evaluated, and H × W represent the spatial resolution of the computational domain. Due to the quadratic nature of its penalty mechanism, RMSE is intrinsically sensitive to large local discrepancies. In the context of highly sparse indoor environments, this characteristic strictly regulates the network to resolve the high-gradient diffusion boundaries of the expanding sneeze cloud, rather than merely converging upon the dominant zero-value background. Fundamentally, RMSE defines the absolute lower bound of the prediction error, verifying how closely the macroscopic scalar field approximates the exact continuum mechanics solution.

#### 2.5.2 Structural similarity: SSIM

While RMSE strictly governs absolute numerical magnitude, it is inadequate for independently assessing the spatial coherence and morphological evolution of the aerosol plume. To evaluate the structural fidelity of the predicted flow topologies, the Structural Similarity Index Measure (SSIM) was introduced. SSIM mathematically models spatial degradation through the formulation presented in Equation 13:

$$SSIM\left(x,y\right)=\frac{\left(2\mu_{x}\mu_{y}+C_{1}\right)\left(2\sigma_{xy}+C_{2}\right)}{\left(\mu_{x}^{2}+\mu_{y}^{2}+C_{1}\right)\left(\sigma_{x}^{2}+\sigma_{y}^{2}+C_{2}\right)}$$ (13)

Where μ and $\sigma^{2}$ represent the empirical mean and variance of the local scalar fields x and y , respectively, and $\sigma_{xy}$ is the cross-covariance. Constants $C_{1}$ and $C_{2}$ are empirically assigned to stabilize the division. Morphologically, SSIM signifies the perceptual and topological congruence between the predicted and true multiphase clouds, quantifying the model’s capacity to maintain intact diffusion boundaries and internal chaotic structural patterns.

#### 2.5.3 Extrema validation: Peak Error

From an epidemiological risk assessment perspective, the maximum localized pathogen concentration fundamentally dictates the upper bound of cross-infection probability. Data-driven models, which predominantly optimize for global averages, frequently exhibit numerical diffusion, artificially smearing the concentration extrema. To rigorously quantify this critical phenomenon, the Peak Error $\left(\epsilon_{\text{peak}}\right)$ is defined in Equation 14 as the relative divergence of the spatial maximums:

$$\epsilon_{\text{peak}}=\frac{1}{N}\overset{N}{\underset{n=1}{\sum }}\frac{\left|\max\left(Y_{\text{pred}}^{\left(n\right)}\right)-\max\left(Y_{\text{true}}^{\left(n\right)}\right)\right|}{\max\left(Y_{\text{true}}^{\left(n\right)}\right)+\epsilon }\times 100\%$$ (14)

Where ϵ is a negligibly small constant to prevent zero division. A minimized Peak Error rigorously suggests that the network successfully resists mode collapse and preserves the transient, high-concentration pathogenic core of the expiratory jet. In practical terms, this metric signifies the model’s reliability in worst-case scenario predictions, explicitly guaranteeing that localized extreme concentration gradients are not artificially smoothed out.

#### 2.5.4 Physics-informed constraints: mass error and CoM distance

To definitively ascertain whether the deep learning predictions implicitly adhere to fundamental fluid mechanics governing laws, two distinct physics-informed metrics were formulated. First, the Mass Error $\left(\epsilon_{\text{mass}}\right)$ evaluates the macroscopic conservation of scalar mass across the computational domain, as defined in Equation 15:

$$\epsilon_{\text{mass}}=\frac{1}{N}\overset{N}{\underset{n=1}{\sum }}\frac{\left|\sum_{i=1}^{H}\sum_{j=1}^{W}Y_{\mathrm{pred}}^{\left(n\right)}\left(i,j\right)-\sum_{i=1}^{H}\sum_{j=1}^{W}Y_{\mathrm{true}}^{\left(n\right)}\left(i,j\right)\right|}{\sum_{i=1}^{H}\sum_{j=1}^{W}Y_{\mathrm{true}}^{\left(n\right)}\left(i,j\right)+\epsilon }\times 100\%$$ (15)

A sub-percent $\epsilon_{\text{mass}}$ mathematically indicates that the network does not arbitrarily hallucinate or annihilate pollutant aerosols, thereby strictly honoring the mass continuity equation.

Furthermore, to assess the network’s capability in capturing advection kinematics, the Center of Mass (CoM) Distance $\left(D_{\text{CoM}}\right)$ was calculated using Equation 16:

$$D_{\text{CoM}}=\frac{1}{N}\overset{N}{\underset{n=1}{\sum }}\sqrt{{\left({\bar{x}}_{\text{pred}}^{\left(n\right)}-{\bar{x}}_{\text{true}}^{\left(n\right)}\right)}^{2}+{\left({\bar{y}}_{\text{pred}}^{\left(n\right)}-{\bar{y}}_{\text{true}}^{\left(n\right)}\right)}^{2}}$$ (16)

Where $\left(\bar{x},\bar{y}\right)$ denote the mass-weighted spatial coordinates of the plume centroid. The CoM Distance provides a stringent geometric quantification of spatial drift. A sub-pixel $D_{\text{CoM}}$ explicitly verifies that the predicted cloud translates in the precise direction and at the exact kinematic speed governed by the Eulerian continuum mechanics. Collectively, these two metrics establish the physical foundation of the surrogate model, ensuring that predictions remain constrained by the laws of physics rather than degenerating into stochastic spatial interpolations.

## 3 Methodology and system implementation

### 3.1 Construction of spatiotemporal CFD dataset

The foundation of the proposed deep learning framework lies in the generation of a high-fidelity, high-volume spatiotemporal dataset that accurately reflects the multiphase flow dynamics of sneeze-induced pollutants.

#### 3.1.1 Simulation case matrix

To ensure the deep learning model possesses sufficient generalization capability across diverse spatial layouts and environmental conditions, a systematic simulation matrix was established. The dataset covers 26 distinct injection configurations, encompassing varying spatial coordinates (x,y) and sneezing orientations to simulate potential infection sources at different bed and desk locations within the dormitory. Each injection configuration was simulated under two distinct ventilation scenarios: a ventilated condition and an unventilated condition, resulting in a total of 52 unique CFD simulation cases.

For each simulation case, the transient evolution of the pollutant concentration field was monitored at two characteristic heights: Z = 1.2 m and Z = 2.0 m. A total of 50 temporal snapshots were extracted for each height per case, generating 5,200 spatiotemporal concentration contour maps in total. The dataset was partitioned into training, validation, and testing sets following an approximate ratio of 7:2:1. Specifically, 3,600 images were allocated for training the network parameters, 1,000 images for hyperparameter tuning and overfitting monitoring during validation, and 600 images were strictly reserved as an unseen testing set to evaluate the final predictive performance.

#### 3.1.2 Mesh independence test

**FIGURE 2:**
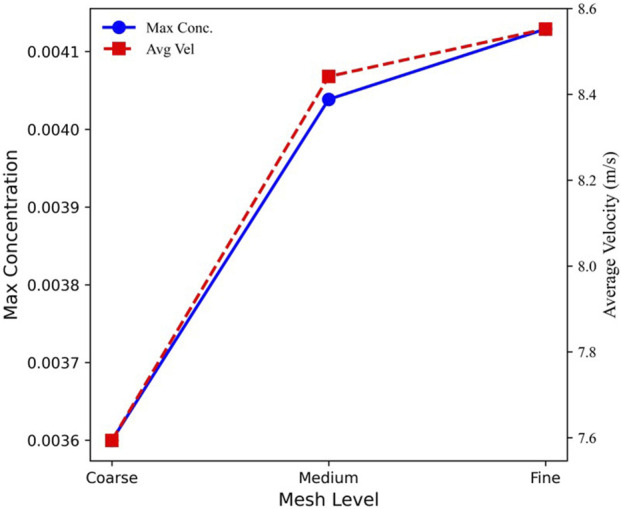
Grid independence test results.

Prior to the execution of batch transient simulations, a rigorous spatial grid independence study was performed to quantify and mitigate grid-induced discretization errors. Three polyhedral mesh configurations with varying spatial resolutions—coarse, medium, and fine—were generated for the dormitory computational domain, with the test results shown in Figure 2.

To evaluate the asymptotic convergence of the numerical solution, the global maximum volume fraction of the pollutant phase and the region-averaged velocity magnitude were selected as the primary monitoring variables. As demonstrated in Figure 2, transitioning from the coarse to the medium mesh induced a significant variation in the monitored flow metrics, indicating inadequate spatial resolution in the coarse configuration. However, as the grid was further refined from the medium to the fine mesh, the scalar and kinematic fields stabilized. Consequently, the medium-density mesh was adopted for all subsequent simulations, providing an optimal trade-off between numerical accuracy and the computational cost required to generate the large-scale dataset.

#### 3.1.3 Raw data acquisition

To transform the physical field data into a format compatible with computer vision algorithms, a standardized protocol for raw data acquisition was implemented in the post-processing stage. First, a Fixed Camera View strategy was strictly enforced. The camera parameters were locked for all 52 cases to ensuring spatial alignment of the dormitory geometry across all images, thereby eliminating geometric distortion or misalignment artifacts. Second, to guarantee that pixel intensity linearly reflects physical quantities, a Fixed Range was applied to the color mapping of the volume fraction contours. The color scale was clamped between a minimum of 0 and a maximum of $8.2\times 10^{-4}$ , preventing the CFD software from auto-scaling the color bar based on local extrema, which would otherwise introduce ambiguity in the pixel-to-concentration mapping. Finally, the data sampling was synchronized with the solver’s time-stepping scheme. The transient snapshots were exported at a high temporal resolution with a time step of 0.01 s, capturing the rapid evolution of the sneeze jet and the subsequent passive diffusion process. This rigorous standardization demonstrates that the input data for the neural network maintains both high physical consistency and visual uniformity.

### 3.2 Dual-stage experimental validation strategy

#### 3.2.1 Stage I: flow field validation

The first stage of validation was conducted to verify the accuracy of the airflow organization predicted by the turbulence model. A high-precision anemometer (Model ST8816A) was selected as the primary measurement instrument, featuring a resolution of 0.001 m/s, which satisfies the sensitivity requirements for detecting low-velocity air currents typical of indoor natural convection. To eliminate measurement errors caused by hand tremors or body heat interference, the anemometer probe was mounted on a rigid tripod stand, strictly maintaining a vertical height of Z = 1.2 m. This height was chosen to align with the breathing zone of occupants in a seated posture, representing the primary domain of interest for potential exposure.

**FIGURE 3:**
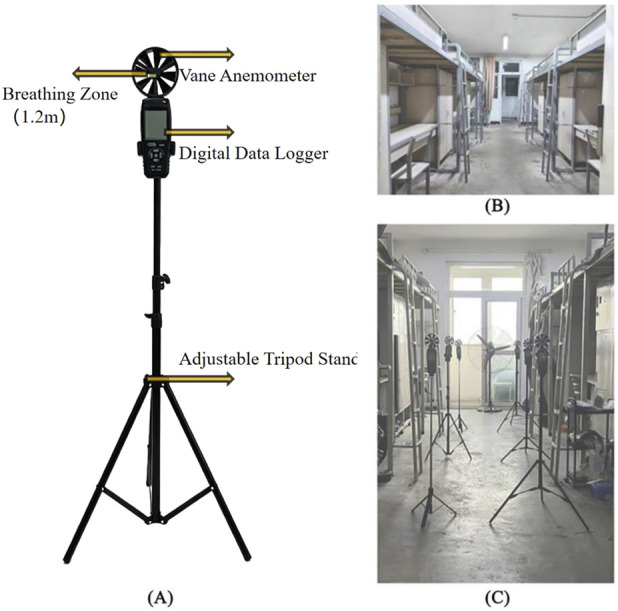
Overview of wind velocity measurement experiments **(A)** composition of the anemometer system **(B)** dormitory test environment **(C)** overview of the experimental site.

An overview of the wind velocity measurement experiments, including the system composition, test environment, and experimental site, is presented in Figure 3.

A spatial array of seven discrete monitoring points was established to characterize the global flow field. Point 0 was positioned immediately adjacent to the air supply inlet to capture the boundary condition velocity. The remaining six measurement points (Points 1–6) were arranged in a structured grid within the main activity zone of the dormitory, with a longitudinal spacing of 1.6 m and a transverse spacing of 0.6 m, ensuring adequate coverage of the velocity gradient regions. For data acquisition, a time-averaged sampling protocol was adopted to filter out transient turbulent fluctuations. At each monitoring point, a continuous sequence of 500 instantaneous velocity readings was recorded under stable environmental conditions. In the post-processing phase, a data cleaning algorithm was applied to filter out anomalous near-zero values attributed to instrumental dead-band noise. The final validation metric for each point was derived by calculating the arithmetic mean of the cleaned time series, which was then compared with CFD simulations to verify the correctness of the flow field configuration.

#### 3.2.2 Stage II: particle trajectory validation

**FIGURE 4:**
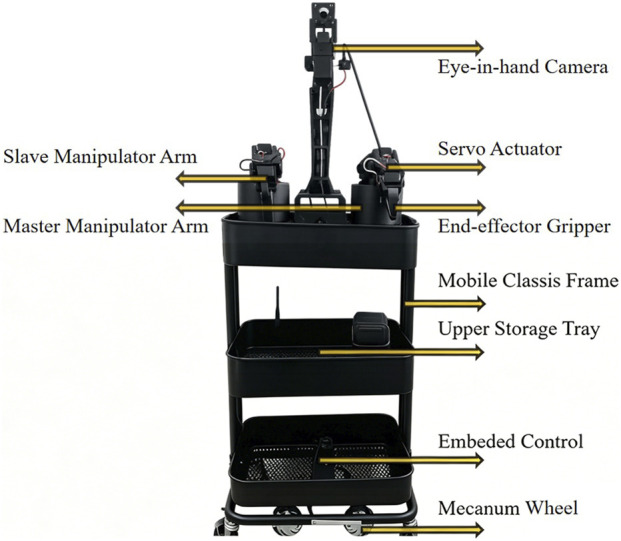
Composition of the robotic arm system.

To allow the human action to freely execute the non-linear physiological sequence, the leader arm was mechanically decoupled from the payload. Specifically, a preparatory cervical extension followed by a large-amplitude forward flexion was implemented. Concurrently, the follower arm, rigidly mounted on an R550 mobile chassis, synchronously mirrored the joint-space maneuvers at a sampling frequency of 30 Hz. Such mobile robotic platforms have proven to be robust tools for indoor environmental monitoring and source localization, offering significantly higher flexibility in spatial sampling compared to stationary sensor arrays. To satisfy the stringent aerodynamic boundary conditions dictated by the CFD Eulerian Mixture Model, it was imperative to synchronize the fluidic ejection with the maximum acceleration point of the robotic torso. During the data acquisition phase, the gripper mechanism (Xie et al., 2023) was logged as a crucial auxiliary action state within the dataset. To prevent over-parameterization while ensuring sufficient stochastic coverage of the action space, 15 spatiotemporally consistent demonstration episodes were recorded under isolated ambient conditions. The overall composition of the robotic arm system, including the master manipulator, end-effector gripper, mobile chassis frame, and mobile chassis components, is illustrated in Figure 4.

The collected multi-modal sensory data, comprising joint positions $q_{t}$ , angular velocities ${\dot{q}}_{t}$ , and the binary gripper action $g_{t}$ were normalized and fed into the Action Chunking with Transformers (ACT) framework. The neural policy was optimized using the AdamW optimizer with an initial learning rate of $1\times 10^{-4}$ and a weight decay of $1\times 10^{-4}$ . Given the explosive nature of the expiratory event, stabilizing the robotic chassis during the downward swing was critical to prevent secondary perturbations. By strictly penalizing the Kullback-Leibler (KL) divergence within the CVAE latent space, the Transformer decoder successfully generated a smooth, jitter-free continuous action chunk ${\hat{A}}_{t}$ of length k=100 steps.

The secondary phase in the Mixture model was defined with a spread parameter and a mean diameter, representing the characteristic polydisperse spectrum of sneeze aerosols. The subsequent 3.53% deviation in the gravitational fallout zone demonstrates that the numerical solver, when initialized with these standardized physiological constants, successfully replicates the discrete particle dynamics observed in the robotic experiment, thereby confirming the framework’s cross-domain reliability.

### 3.3 Data preprocessing pipeline

Raw simulation data exported from CFD software often contains noise and redundant information that can hinder model convergence. To construct a high-quality dataset for the deep learning model, a rigorous preprocessing pipeline was developed.

#### 3.3.1 ROI extraction and spatial normalization

The raw concentration contour images obtained from ANSYS Fluent typically include non-physical elements such as white backgrounds, legends, and axes. To extract the pristine aerodynamic domain autonomously, a robust morphological alignment pipeline (BlueLockCrop) was implemented. By converting the RGB matrices into the Hue-Saturation-Value (HSV) color space, the continuous fluidic region was isolated via dynamic color thresholding. Subsequently, a morphological closing operator utilizing a large spatial kernel was applied to obliterate microscopic grid-point anomalies and bridge numerical voids, rendering a continuous and solid Region of Interest (ROI). Following boundary box extraction, the resulting tensors were strictly resampled to a resolution of 640 × 320 pixels. This specific resolution is not arbitrary; it is mathematically mandated by the spatial-preserving constraints of the U-ConvLSTM architecture. Since the symmetric encoder incorporates four sequential strided downsampling operations, the input dimensionality must be perfectly divisible by $2^{4}=16$ . Adhering to this rigorous dimension mitigates sub-pixel interpolation drift during transposed upsampling, preventing non-physical vertical advection over long-term autoregressive predictions.

#### 3.3.2 Dual-channel input construction

Unlike conventional single-layer image classification tasks, pollutant dispersion within a dormitory exhibits complex vertical interactions driven by thermal stratification and gravitational settling. To capture this vertical coupling effect, a Dual-Channel Input Strategy was developed. For each time step t , normalized concentration fields are extracted from two distinct height planes: the lower breathing zone $Z_{\text{low}}=1.2$ m and the upper breathing zone $Z_{\text{high}}=2$ m. Rather than treating these as independent samples, the two concentration maps are stacked along the channel dimension to form a unified 3D input tensor $X_{t}\in R^{H\times W\times 2}$ . Finally, pixel values are normalized to the range [0,1] to stabilize gradient descent optimization during model training.

#### 3.3.3 Sliding window sequence generation

To enable supervised learning for the time-series forecasting task, the continuous CFD data is transformed using a Sliding Window Strategy. Let the complete time series of a simulation case be $X=\left\{X_{1},X_{2},\ldots ,X_{N}\right\}$ . We define an input historical window $T_{\text{in}}=5$ and a prediction horizon $T_{\text{out}}=1$ . The dataset is constructed by sliding this window with a stride of one across the temporal dimension. This process generates samples where the input is a sequence of 5 consecutive dual-channel frames $\left\{X_{t},\ldots ,X_{t+4}\right\}$ and the target is the subsequent frame $X_{t+5}$ . This strategy not only significantly expands the volume of the training dataset but also enables the model to learn dispersion dynamics at various stages, from the initial high-momentum jet to the later passive diffusion phase.

### 3.4 Deep learning model implementation

The proposed framework is implemented using the PyTorch deep learning library. To ensure the reproducibility of the results, the specific configurations of the network architecture and the design of the objective function are detailed below.

#### 3.4.1 Network architecture configuration

The hybrid model follows an Encoder-Recurrent-Decoder paradigm, the input to the model is a tensor of shape $\left(B,T_{\text{in}},C,H,W\right)$ , where B is the batch size, $T_{\text{in}}=5$ is the sequence length, C=2 represents the dual-height channels, and H×W=640×320 . To process the temporal sequence, a TimeDistributed wrapper is applied to the CNN backbone. This mechanism demonstrates that the same set of convolutional weights is shared across all time steps. Rather than collapsing the spatial topology through adaptive pooling, multiple strided convolutions are utilized to compress the spatial features, independently extracting a localized spatial feature tensor $V_{t}$ for each frame. The output feature maps with a channel size $D_{\text{model}}=256$ to serve as the input for the recurrent layer.

Subsequently, to capture the non-linear temporal dynamics of pollutant diffusion, a multi-layer ConvLSTM structure is employed. The hidden state dimension is set to 512 to retain sufficient memory of the flow history. A stacked architecture with 2 ConvLSTM layers is utilized, where the first layer captures low-level temporal dependencies and the second layer models high-level evolution trends. To prevent overfitting on the training data, a dropout rate of 0.2 is applied between the recurrent layers.

Finally, the Upsampling Decoder reconstructs the predicted feature vector h t+1 back into the spatial domain. This is achieved by symmetric blocks of Transposed Convolutional Layers, Batch Normalization, and ReLU operations, which progressively upsample the resolution. To faithfully recover the original spatial dimensions and fine-grained dispersion boundaries, skip-connections are subsequently applied to concatenate encoding features directly to the final target resolution of 640 × 320. A Sigmoid activation function is applied at the final layer to bound the predicted pixel values within the normalized range of [0,1], ensuring physical consistency with the concentration mass fraction.

#### 3.4.2 Loss function design

In standard regression tasks for fluid dynamics, relying solely on a uniform Mean Squared Error (MSE) objective frequently induces severe numerical diffusion. This occurs because the extreme spatial sparsity inherent in indoor CFD fields biases the gradient descent towards optimizing the background, thereby heavily attenuating the critical high-concentration pathogenic core.

To rigorously enforce both pixel-wise numerical accuracy and macroscopic structural fidelity, a sophisticated physics-informed composite loss function $\left(L_{\text{total}}\right)$ was mathematically formulated. The total objective integrates four complementary penalty mechanisms, as defined in Equation 17:

$$L_{\mathrm{total}}=\lambda_{\mathrm{MSE}}\cdot L_{\mathrm{MSE}}^{\Omega }+\lambda_{L1}\cdot L_{L1}^{\Omega }+\lambda_{\mathrm{SSIM}}\cdot L_{\mathrm{SSIM}}+\lambda_{\mathrm{peak}}\cdot L_{\mathrm{peak}}$$ (17)

To specifically penalize numerical diffusion within the sparse sneeze plume, an adaptive spatial weighting matrix Ω(x,y) was introduced into the focal reconstruction losses ( $L_{\mathrm{MSE}}^{\Omega }$ and $L_{L1}^{\Omega }$ ). The weight scales linearly up to a factor of $\lambda_{\mathrm{cloud}}=20.0$ , activated strictly by the presence of ground-truth concentration vectors, as defined in Equation 18:

$$\Omega \left(x,y\right)=1.0+\left(\lambda_{\mathrm{cloud}}-1.0\right)\cdot \max_{c\in \left\{R,G\right\}}Y_{\mathrm{true}}^{\left(c\right)}\left(x,y\right)$$ (18)

Consequently, the spatially-weighted reconstruction losses are defined in Equation 19:

$$L_{\mathrm{MSE}}^{\Omega }=\frac{\sum \left({\left(Y_{\mathrm{pred}}-Y_{\mathrm{true}}\right)}^{2}⊙\Omega \right)}{\sum \Omega },\;L_{L1}^{\Omega }=\frac{\sum \left(|Y_{\mathrm{pred}}-Y_{\mathrm{true}}|⊙\Omega \right)}{\sum \Omega }$$ (19)

Where ⊙ denotes the Hadamard element-wise product. The inclusion of the weighted L1 norm forcefully suppresses background noise and sharpens the dispersion boundaries.

Furthermore, the Structural Similarity Loss preserves the non-linear topological morphology of the turbulent cloud. Furthermore, a scalar peak conservation term $\left(L_{\text{peak}}\right)$ was incorporated to guarantee that the absolute maximum predicted pathogenic payload matches the ground-truth extrema, and is defined in Equation 20 as:

$$L_{\mathrm{peak}}=|\max\left(Y_{\mathrm{pred}}\right)-\max\left(Y_{\mathrm{true}}\right)|$$ (20)

Through systematic hyperparameter optimization, the balancing coefficients were empirically finalized as $\lambda_{\mathrm{MSE}}=1.0$ , $\lambda_{L1}=1.0$ , $\lambda_{\mathrm{SSIM}}=0.5$ , $\lambda_{\mathrm{peak}}=1.0$ . This composite formulation prioritizes the exact localization of extreme spatial gradients while simultaneously maintaining the continuous Eulerian continuum topology.

#### 3.4.3 Bayesian hyperparameter optimization framework

Translating the highly non-linear theoretical loss landscape $\left(L_{\text{total}}\right)$ into a stable and convergent computational pipeline heavily relies on the precise configuration of learning parameters. Given the extreme spatial sparsity inherent in indoor CFD prediction, heuristic or manual tuning of the network typically risks mode collapse or gradient explosion. To systematically mitigate this instability, a sophisticated Bayesian hyperparameter optimization framework was deployed leveraging the Optuna library.

The optimization objective was strictly defined to minimize the validation loss by iteratively exploring the posterior probability distribution within a multi-dimensional continuous search space. The targeted search parameters included the base learning rate, the batch size, and most importantly, the dynamic focal penalty weight for the high-concentration aerosol cloud. The exploration was driven by the Tree-structured Parzen Estimator (TPE) algorithm, which effectively models the expected improvement of hyperparameter combinations over sequential trials.

To substantially accelerate the computational search process and ensure optimal resource allocation, a MedianPruner early-stopping protocol was integrated. This algorithm actively monitored the learning curve during the initial epochs and prematurely terminated unpromising trials that exhibited early symptoms of mean-color collapse. After 30 independent systematic trials, the sensitivity analysis identified the optimal configuration capable of suppressing background bias while preserving sharp advection gradients. The finalized model adopted $\lambda_{\mathrm{cloud}}=20.0$ , a batch size of 4, and an Adam optimizer. Furthermore, a learning rate warm-up strategy was implemented over the initial 5 epochs to stabilize the randomly initialized weights, which subsequently transitioned to a ReduceLROnPlateau scheduler to guarantee smooth asymptotic convergence towards the complex multiphase fluid dynamics.

### 3.5 Epidemiological risk quantification and envelope analysis

#### 3.5.1 Tensor-to-euclidean inverse mapping and threshold calibration

To translate the highly abstract scalar predictions into practical public health engineering metrics, an automated post-processing algorithm was constructed to execute an inverse geometric projection. The discrete concentration tensors output by the U-ConvLSTM were mathematically mapped back into the continuous physical Euclidean space corresponding to the dormitory dimensions. Consequently, an absolute spatial calibration was established, translating the tensor coordinate system into standard metric units ( Δx≈0.0113 m/pixel, Δy≈0.0106 m/pixel).

Following the spatial calibration, it was imperative to differentiate the highly infectious pathogenic core from negligible numerical diffusion. Aligning with the fundamental principles of the Wells-Riley infection probability model, the critical pathogenic threshold $\left(\tau_{\text{crit}}\right)$ for the expiratory plume was empirically calibrated at 5% of the maximum normalized ejection concentration. Filtering the raw tensor output through this stringent threshold explicitly eradicates insignificant background fluctuations and ambient noise. This truncation mechanism exclusively isolates the high-momentum multiphase core, which is the absolute prerequisite for evaluating the worst-case cross-infection boundaries without overestimating the diffusion volume.

#### 3.5.2 Mathematical formulation of the dynamic safety radius

Unlike conventional static distancing protocols, expiratory events in confined unventilated spaces exhibit a highly transient, non-linear spatiotemporal expansion. To dynamically quantify this risk, the Dynamic Safety Radius $\left(D_{\text{safe}}\left(t\right)\right)$ was mathematically formulated. Let Φ(x,y,t) denote the normalized concentration field predicted at the horizontal breathing plane. For any specific time step t , the hazardous spatial domain $\Omega_{t}$ is rigorously defined by isolating all Eulerian coordinates (x,y) where the local mass fraction strictly exceeds the aforementioned critical threshold $\tau_{\text{crit}}$ as shown in Equation 21:

$$\Omega_{t}=\left\{\left(x,y\right)\in R^{2}|\Phi \left(x,y,t\right)\geq \tau_{\mathrm{crit}}\right\}$$ (21)

Subsequently, rather than relying on arbitrary linear measurements, the dynamic safety radius is quantified as the maximum Euclidean distance from the sneeze origin $p_{s}=\left(x_{s},y_{s}\right)$ to the extreme boundary of the hazardous domain $\Omega_{t}$ as defined in Equation 22:

$$D_{\mathrm{safe}}\left(t\right)=\max_{p\in \Omega_{t}}\|p-p_{s}{\|}_{2}$$ (22)

This continuous geometric formulation accommodates the asymmetric nature of turbulent advection, enabling a precise, localized quantification of the expanding infection perimeter.

#### 3.5.3 Global statistical envelope computation

A fundamental limitation of deterministic Computational Fluid Dynamics is its confinement to specific release scenarios, making it computationally prohibitive to derive generalized epidemiological guidelines. Empowered by the $O\left(10^{3}\right)$ real-time acceleration of the U-ConvLSTM surrogate, this study implemented a global statistical envelope algorithm to perform a probabilistic risk assessment across the entire dormitory.

An automated pipeline was programmed to systematically iterate over spatial sneeze configurations. For each configuration, the algorithm calculated the instantaneous $D_{\text{safe}}\left(t\right)$ over a 50 steps trajectory. By aggregating these individual trajectories into a systematic spatiotemporal matrix, the algorithm computed the ensemble mean, alongside the absolute minimum and maximum bounds at each time step. The resulting Statistical Envelope delineates the worst-case and best case expansion limits governed by diverse wall impingement effects and convective orientations. This stochastic methodology definitively upgrades the safety distance analysis from a localized observation to a generalized, highly robust probabilistic framework, providing a theoretical foundation for dynamic spatial layout optimization.

## 4 Results and discussions

### 4.1 Dual-stage experimental validation analysis

#### 4.1.1 Flow field validation (stage I)

**FIGURE 5:**
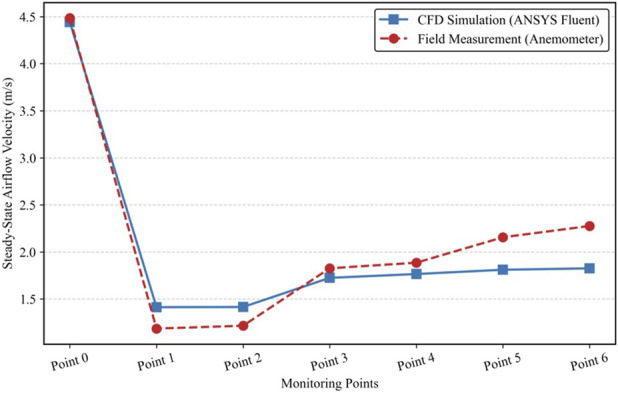
Comparison between simulation and measurement of wind velocity.

The validation of the continuous phase flow field lays the aerodynamic foundation for subsequent Lagrangian particle tracking. A quantitative comparison between numerically resolved velocity magnitudes and experimental anemometric data at seven distinct monitoring locations, as presented in Figure 5 and Table 2, verifies the predictive capability of the turbulence model. As shown in the measurement results, airflow velocities in the domain range from approximately 1.18 m/s to 4.49 m/s, representing a transition from the high-momentum jet region to the mixed convection zone.

**TABLE 2:** Comparison of simulated and measured airflow velocities.

Monitoring	Simulated	Measured	Relative error
Point	Velocity (m/s)	Velocity (m/s)	(%)
Point 0	4.4437	4.4856	−0.93
Point 1	1.4127	1.1855	19.16
Point 2	1.4145	1.2153	16.39
Point 3	1.7244	1.8259	−5.56
Point 4	1.7649	1.8853	−6.39
Point 5	1.8103	2.1555	−16.01
Point 6	1.8251	2.2755	−19.79

At the inlet region (Point 0), the numerical model exhibits high fidelity, with the simulated velocity closely matching the experimental value, yielding a relative error of merely −0.93%. This negligible deviation confirms that the boundary conditions for the air supply were correctly imposed and the high-Reynolds-number jet behavior was accurately resolved. For the interior measurement points (Points 1-6), located in the downstream region characterized by complex recirculation and velocity decay, the simulation captures the overall airflow trends, though local discrepancies increase. The relative errors in these regions fluctuate between 5.56% and 19.79%.

According to the validation framework for indoor environment CFD analyses established by Chen and Srebric, predicting low-velocity indoor airflow poses a significant challenge due to the coexistence of laminar, transitional, and turbulent regimes. Chen and Srebric noted that for such complex mixed-convection flows, discrepancies in the range of 10%–20% are consistent with the inherent limitations of RANS formulations and are considered acceptable for engineering applications, provided the global flow patterns are correctly captured. Furthermore, the positional uncertainty of the probe and minor thermal plumes from the experimental setup may also contribute to the local variance.

Despite the point-wise deviations, the global statistical metrics demonstrate the robustness of the numerical model. To systematically evaluate the agreement between the simulated and measured datasets, the Pearson correlation coefficient (r) , Normalized Mean Square Error (NMSE), and Fractional Bias (FB) were calculated. The analysis yields a correlation coefficient of r=0.9744 , indicating a strong linear relationship and consistent flow topology between simulation and experiment. Furthermore, the NMSE value of 0.059 and the Fractional Bias (FB) of −0.043 fall well within the rigorous acceptance criteria for indoor environmental modeling (typically NMSE<0.25 and |FB|<0.5 ). Consequently, based on both trend consistency and statistical validation metrics, the simulated flow field is considered to possess sufficient physical fidelity to serve as the carrier phase for the subsequent droplet dispersion study.

The observed gradient in relative error from a high-fidelity 5.56% in the jet-dominated regions to 19.79% in the complex recirculation zones reflects the spatial non-uniformity of RANS turbulence closure performance. While the lower bound of this range surpasses the typical 10%–20% accuracy threshold cited in literature, the upper-bound variance presents a potential source of uncertainty propagation for the surrogate model.

The quantitative impact of this validation error on the U-ConvLSTM’s predictive performance manifests primarily as a spatial-temporal shift in the underlying fluidic manifold. Since the neural network is trained to emulate the Eulerian-Eulerian Mixture solutions, the velocity field discrepancy in Stage I directly modulates the convective transport terms learned by the model. Specifically, the 19.79% error in complex recirculation zones implies that while the network can achieve high numerical precision relative to its CFD training baseline, its absolute predictive fidelity when mapped back to real-world coordinates inherits this physical uncertainty.

However, a critical distinction lies in the decoupling of topological integrity from advective velocity variance. While the 10%–20% velocity error may induce a proportional phase lag or lead in the pollutant cloud’s longitudinal displacement, it does not degrade the network’s ability to resolve the sharp concentration gradients and multi-scale turbulent structures. The spatial-preserving U-Net encoder, stabilized by skip-connections, ensures that even when the underlying velocity field exhibits numerical bias, the absolute physical coordinates (x,y) of the pollutant core remain structurally consistent.

#### 4.1.2 Particle trajectory validation via robotic arm (stage II)

**FIGURE 6:**
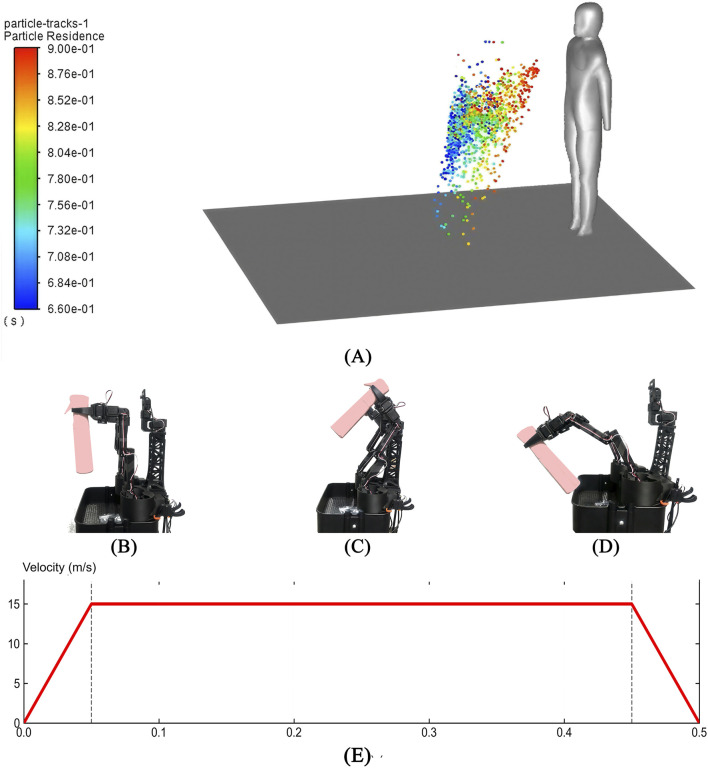
Overview of the robotic arm and simulation process **(A)** particle dispersion behavior after sneezing **(B)** robotic arm simulating the stationary state **(C)** robotic arm simulating the backward-leaning state **(D)** robotic arm simulating the ejection state **(E)** acceleration–peak–decay cycle.

To strictly validate the physical fidelity of the Eulerian-Eulerian multiphase solver in capturing the transient expiration dynamics, a full-scale anthropomorphic experiment was deployed within a structurally identical dormitory environment. As illustrated in Figure 6, the SO-ARM101 robotic twin, conditioned by the Imitation Learning policy, was positioned at the exact spatial coordinates defined in the CFD domain Z = 1.2 m.

**FIGURE 7:**
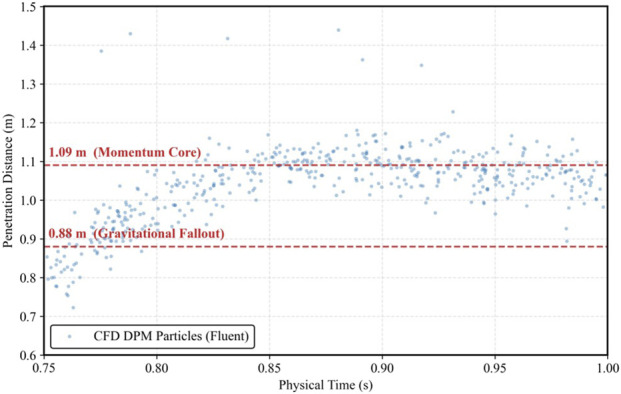
Scatter plot of simulated droplet distribution distances.

Figure 7 illustrates the temporal evolution of the particle penetration distance predicted by the CFD solver between t = 0.75 s and 1.0 s. The numerical scatter plot reveals a distinct aerodynamic bifurcation: the bulk of the multiphase cloud stabilizes within a penetration plateau ranging from 0.80 m to 1.15 m around t = 0.85 s, constrained by the exponential decay of the initial ejection momentum and subsequent turbulent dispersion.

The experimental measurements successfully captured two representative maximal impingement boundaries: 0.88 m and 1.09 m. As summarized in Table 3, an in-depth physical traceability analysis was conducted to correlate these discrete experimental marks with the continuous numerical distribution. The measurement at 1.09 m exhibits an exceptional congruence yielding a relative deviation of −2.68% with the dense momentum core of the CFD prediction 1.12 m. This rigorously substantiates that the numerical solver accurately captures the dominant advection-diffusion kinetics of the primary aerosol cloud.

**TABLE 3:** Quantitative validation of droplet penetration distances.

Droplet regime	Experimental	CFD	Relative deviation
	(m)	Prediction(m)	(%)
Gravitational fallout	0.88	0.85	3.53
Momentum core	1.09	1.12	−2.68

Furthermore, the 0.88 m measurement perfectly aligns with the lower boundary of the CFD cluster 0.85 m, yielding a relative deviation of 3.53% . From a fluid mechanics perspective, this corresponds to the gravitational fallout regime. It indicates that a specific sub-population of highly agglomerated, massive droplets rapidly decouples from the horizontal airstream due to gravity dominance, settling prematurely before reaching the maximum penetration depth.

Consequently, this validated numerical baseline provides a fundamentally reliable and dimensionally accurate training manifold for the subsequent U-ConvLSTM surrogate model, ensuring that the deep learning predictions are anchored in physical reality.

### 4.2 Predictive performance assessment

**FIGURE 8:**
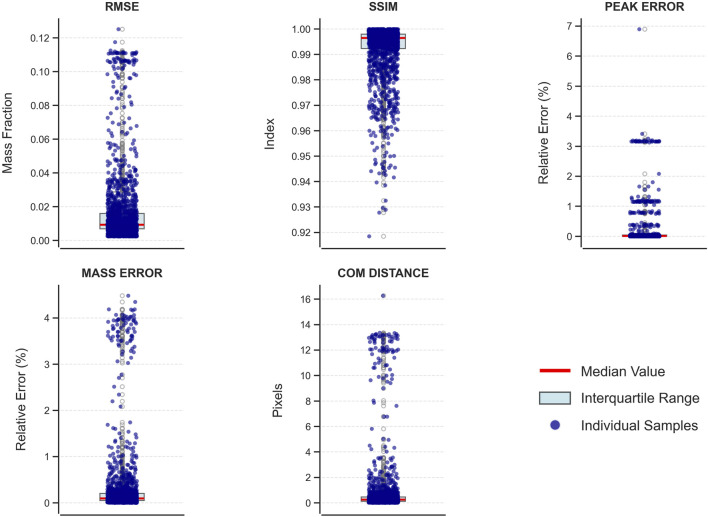
Boxplot of quantitative indicators for prediction results.

To quantify the generalization capability and hydrodynamic fidelity of the proposed U-ConvLSTM model, an exhaustive statistical evaluation was conducted across the isolated testing set. Moving beyond conventional global error metrics, the performance assessment incorporated topological and physics-informed metrics to evaluate the aerosol dispersion characteristics systematically. As visualized in Figure 8, the global statistical distributions of the five designated evaluation metrics are presented via box-and-swarm plots, with their corresponding mathematical expectations summarized as follows: RMSE = 0.0167, SSIM = 0.9923, Peak Error = 0.1356, Mass Error = 0.3135%, and CoM Distance = 0.888 pixels.

First, evaluating the fundamental numerical accuracy and structural topology, the model achieved a substantially low RMSE (0.0167) and a near-optimal SSIM (0.9923). As visually corroborated by the dense swarm distribution, the interquartile ranges for both metrics are exceedingly narrow. This high structural similarity confirms that the purely convolutional architecture intrinsically preserves the spatial advection boundaries without the topological destruction typically induced by fully connected layers. However, it is critical to acknowledge that the exceptionally high SSIM is partially attributable to the extreme spatial sparsity of indoor CFD domains, where the unpolluted ambient background predominates.

Therefore, relying solely on global pixel-wise averages masks the network’s capability to resolve the critical pathogenic core. To this end, Mass Error and Center of Mass (CoM) Distance provide a more stringent evaluation. The U-ConvLSTM model yielded a macroscopic Mass Error of merely 0.3135%. This sub-percent discrepancy suggests that the surrogate model implicitly adheres to the Eulerian continuity equation, actively preventing the non-physical generation or annihilation of aerosol particles during the temporal rollout. Concurrently, the CoM Distance averaged 0.888 pixels. A spatial drift of less than a single pixel validates that the predicted plume tracks the advection velocity and thermodynamic trajectory governed by the numerical solver.

Furthermore, in epidemiological engineering applications, the worst-case scenario dictates the upper bound of infection risk. The network achieved a relative Peak Error of 13.56%. Unlike the highly concentrated interquartile ranges of the RMSE and SSIM, the swarm plot for Peak Error exhibits a broader variance with several noticeable outliers. From a fluid dynamics perspective, this significant standard deviation is a reflection of the highly non-linear nature of turbulent diffusion. Specifically, during the initial explosive phase of the sneeze or when the jet impinges upon the rigid boundaries, the local concentration gradients undergo drastic fluctuations. The broader distribution in Peak Error accurately mirrors the network’s proactive effort to reconstruct these transient, high-frequency aerodynamic collisions, rather than defaulting to a uniform, smoothed mean-color mapping. Overall, the systematic metric statistics verify that the proposed model satisfies both numerical precision and stringent physical conservation laws.

### 4.3 Spatiotemporal visualization of predictive performance

#### 4.3.1 Qualitative superiority over conventional architectures

**FIGURE 9:**
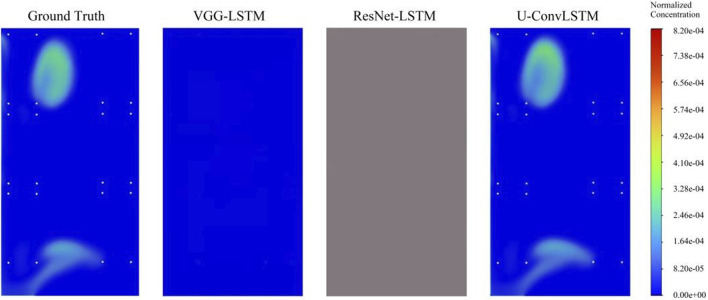
Comparison of prediction between the U-ConvLSTM model and existing models.

To fundamentally substantiate the structural necessity of the proposed purely convolutional U-ConvLSTM, a qualitative comparative analysis was conducted against established classification-based spatial encoders. As depicted in Figure 9, given identical historical flow fields, the predictions generated by the conventional architectures exhibited catastrophic aerodynamic distortion.

Specifically, the VGG-LSTM model demonstrated severe background bias, predicting a completely uniform zero-value domain. This failure stems from the extreme spatial sparsity of indoor CFD environments. The flattening operations in VGG indiscriminately averaged the sparse high-concentration pixels with the dominant background, causing the network to converge onto a solution that eradicated the pathogenic sneeze core. Conversely, the ResNet-LSTM suffered from profound mean color collapse, rendering a non-physical, homogenous gray scalar field. While residual connections facilitate deep feature propagation, the terminal Global Average Pooling layer obliterates the spatial coordinates (x,y). Consequently, the network recognized the existence of the multiphase plume but completely lost its spatial frame of reference, forcing the decoder to distribute the concentration uniformly across the entire domain to minimize global variance.

In stark contrast, the proposed U-ConvLSTM strictly bypasses vector flattening. By maintaining the 4D tensor structure (B,C,H,W) and leveraging symmetric skip-connections, our model successfully retained the absolute Eulerian coordinates. As shown in the comparison, it accurately reconstructed the sharp, localized concentration gradients of the expiratory jet, demonstrating that preserving topological architecture is an absolute prerequisite for high-fidelity fluid dynamics forecasting.

#### 4.3.2 High-fidelity contour reconstruction and error topology analysis

**FIGURE 10:**
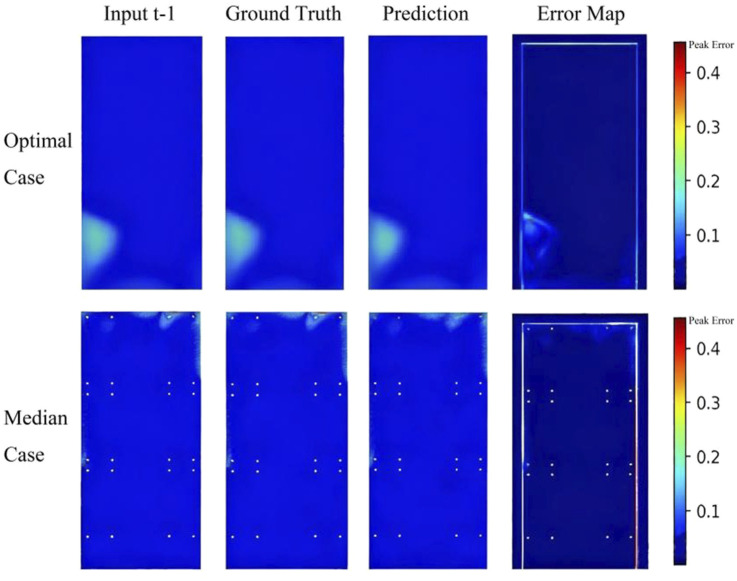
Prediction error map.

Beyond comparing with baselines, an in-depth spatiotemporal error traceability analysis was performed to evaluate the physical robustness of the U-ConvLSTM model. Figure 10 illustrates the input, ground truth, prediction, and absolute error map for two representative scenarios: an optimal case and a median case.

Visual inspection confirms that the predicted aerosol plumes exhibit remarkable morphological congruence with the CFD numerical solutions. The network accurately captured the non-linear expansion and the convective trajectory of the sneeze jet. More importantly, the Error Maps reveal that the numerical deviations are not randomly distributed but follow a distinct physical and geometric topology. The residual errors are predominantly localized at the highly turbulent leading edges of the plume and the impingement zones near the rigid wall boundaries. From a fluid mechanics perspective, these regions are characterized by intense scale momentum mixing and extremely sharp concentration gradients, which inherently amplify interpolation uncertainty during the network’s transposed convolution phase.

Furthermore, as observed in the median loss scenario, distinct linear error artifacts appear exactly along the structural boundaries of the dormitory. This is a classic manifestation of the zero padding mechanism in Convolutional Neural Networks, which struggles to perfectly replicate the non-slip boundary conditions of finite domains. Despite these highly localized boundary and interfacial artifacts, the overall scalar conservation and the trajectory of the main pathogenic core remain remarkably intact. This topology analysis substantiates that the model successfully prioritizes macroscopic mass transport over high-frequency local turbulent fluctuations, proving its reliability for indoor micro-environmental risk assessment.

#### 4.3.3 Hierarchical feature extraction mechanics

**FIGURE 11:**
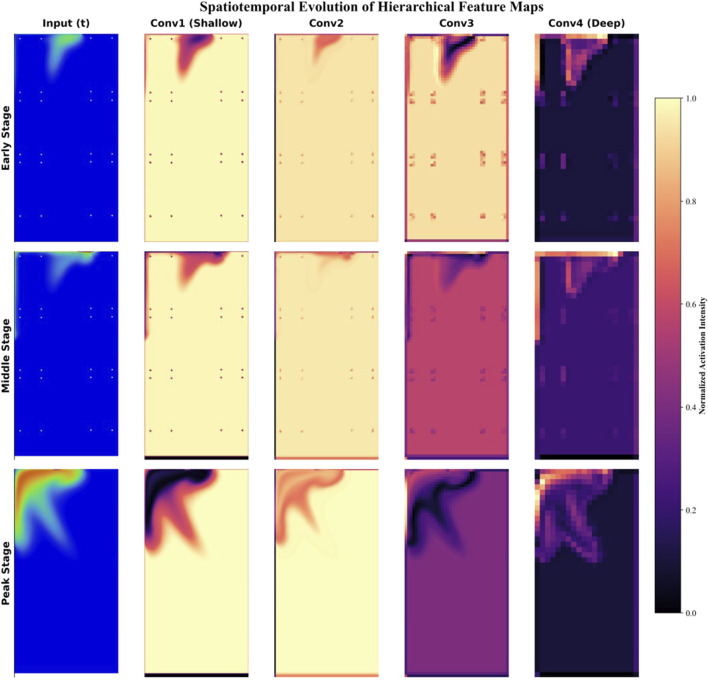
Convolutional layer visualization.

The intermediate latent representations of the internal encoding mechanics of the proposed U-ConvLSTM architecture were visualized. Figure 11 illustrates the spatiotemporal evolution of the feature maps extracted from four consecutive convolutional layers across three distinct aerodynamic stages of the sneeze event. By normalizing and averaging the high-dimensional channel activations, the network’s hierarchical logic in decoding multi-phase fluid dynamics is explicitly revealed.

The convolutional hierarchy exhibits a functional transition from high-frequency spatial gradient detection to low-frequency macroscopic momentum encapsulation. In the shallow layer, the activation heatmaps predominantly highlight the sharp multiphase interfaces and the turbulent leading edges of the aerosol plume. It should be noted that Conv1 also aggressively captures static, high-contrast background artifacts, confirming that shallow filters are universally sensitive to local pixel-level variations.

Conversely, as the tensors traverse the spatial bottlenecks into the deeper layers, the spatial resolution is progressively downsampled by a factor of 16, compelling the network to discard redundant geometrical noise. This abstractive mechanism is most pronounced in the Conv4 feature maps. It is observed that the non-physical static background markers are completely attenuated in the deep layers. Instead, the profound activations in Conv4 are exclusively locked onto the macroscopic aerodynamic momentum core of the sneeze jet.

Furthermore, from a temporal perspective, the feature maps dynamically adapt their activation topologies to track the expanding trajectory of the bio-aerosol cloud. This spatiotemporal coherence rigorously suggests that the purely convolutional encoder does not merely memorize static images; rather, it effectively isolates the pathogenic kinetic energy and inherently learns the underlying advection-diffusion kinetics without requiring explicit Eulerian governing equations.

### 4.4 Computational efficiency and engineering application

#### 4.4.1 Computational efficiency and real-time inference acceleration

Beyond topological fidelity and numerical precision, the fundamental impetus for developing data-driven surrogates resides in circumventing the severe computational latency inherent in conventional numerical solvers. To quantitatively evaluate the acceleration performance, a computational cost analysis was conducted, comparing the traditional transient CFD simulation against the proposed U-ConvLSTM inference on identical hardware architectures for generating a single scalar contour frame.

**FIGURE 12:**
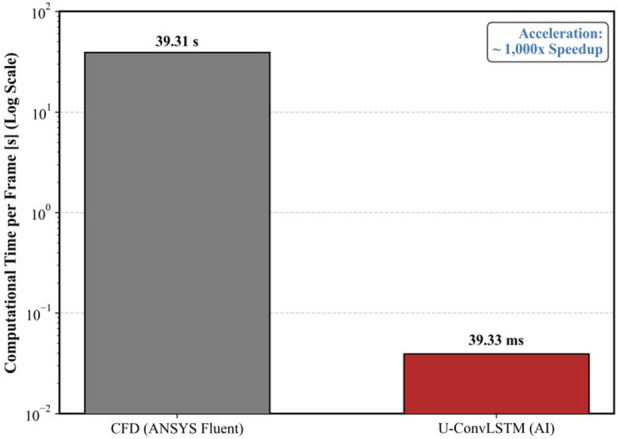
Comparison of time between deep learning model and ANSYS.

As illustrated in Figure 12, utilizing the Eulerian-Eulerian multiphase model in ANSYS Fluent required an average of 39.31 s on a high-performance commercial workstation. This latency originates from the fundamental mechanics of CFD solvers: capturing the highly non-linear multiphase transport and turbulent diffusion necessitates iterative convergence of the Navier-Stokes equations at every microscopic time step, strictly constrained by the Courant-Friedrichs-Lewy (CFL) condition. Consequently, accumulating these frame-wise delays renders conventional CFD entirely unfeasible for real-time dynamic risk mapping.

Conversely, the proposed U-ConvLSTM model fundamentally bypasses the iterative numerical integration process. By mapping the spatiotemporal evolution into a feed-forward tensor matrix multiplication, the trained neural network achieved an average inference latency of merely 39.33 m per frame. This translates to an extraordinary computational acceleration of three orders of magnitude.

From an engineering perspective, reducing the processing time from tens of seconds to the millisecond regime represents a paradigm shift. It signifies that the proposed AI surrogate successfully transitions high-fidelity bio-aerosol forecasting from a computationally intensive, retrospective offline task into an instantaneous, deployable tool. This $O\left(10^{3}\right)$ magnitude speedup fulfills the fundamental prerequisite for integrating dynamic safety distance algorithms with active control systems, enabling instantaneous epidemic prevention responses in confined densely populated spaces.

#### 4.4.2 Global safety envelope and dynamic risk quantification

A fundamental limitation of deterministic CFD studies is their inherent confinement to specific, isolated release scenarios. This computational inflexibility renders the derivation of generalized epidemiological guidelines virtually unattainable. Empowered by the real-time inference acceleration of the proposed U-ConvLSTM surrogate, this study executed a systematic statistical envelope analysis across diverse spatial orientations and bed locations, comparing unventilated and mechanically ventilated dormitory environments.

**FIGURE 13:**
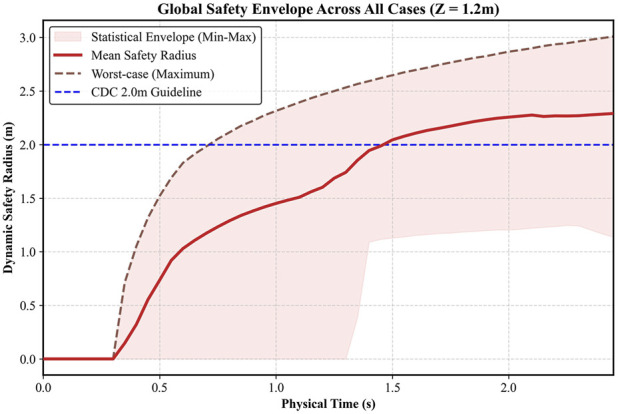
Statistical envelope of the Safe Distance in unventilated conditions.

The spatiotemporal evolution of the hazardous radius exhibits a distinct divergence in aerodynamic lifecycles. As illustrated in Figure 13, the trajectory of the mean Dynamic Safety Radius $\left(D_{\text{safe}}\right)$ expands aggressively during the initial momentum-driven phase, breaching the conventional CDC 2.0-m social distancing guideline at t≈1.5 s and peaking at 2.26 m. The upper boundary of the shaded envelope, representing the absolute worst-case scenario governed by unobstructed release orientations, reaches a maximum radius of 3.30 m within 1.8 s. In the absence of external momentum flux, the pollutant cloud is primarily governed by passive turbulent diffusion and thermal buoyancy, leading to the formation of a stagnant pollutant reservoir that lingers within the breathing zone. This demonstrates that static 2.0-m distancing protocols are fundamentally inadequate and structurally vulnerable in unventilated, densely occupied confined spaces.

**FIGURE 14:**
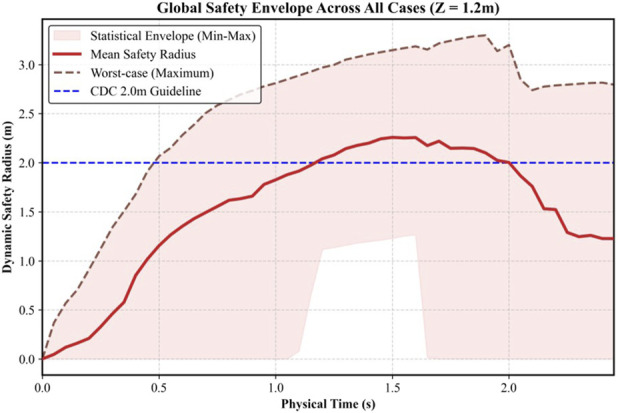
Spatiotemporal evolution of Safe Distance under active mechanical ventilation.

In contrast, the introduction of mechanical ventilation fundamentally alters the plume topology and risk duration. As illustrated in Figure 14, while the initial expansion is marginally accelerated crossing the 2.0 m threshold at t≈1.2 s due to the superposition of the sneeze jet and the background airflow. Beyond t≈1.6 s, the mean $D_{\text{safe}}$ transitions into a marked contraction trajectory, successfully receding below the 2.0 m threshold by t≈2.0 s. From a fluid dynamics perspective, this decline signifies a critical aerodynamic transition where the forced convection induced by the pressure gradient overrides the momentum-dominated advection.

As the expiratory plume expands volumetrically, the localized mass fraction is heavily diluted by the continuous entrainment of ambient air. Ultimately, this topological dissipation confirms that the paramount risk of direct acute droplet impingement is strictly concentrated within a transient critical exposure window under ventilated conditions. Whereas unventilated spaces present a persistent spatial threat, strategic ventilation transforms the infection risk into a manageable temporal event, necessitating the integration of dynamic distancing and active HVAC control in university dormitories.

## 5 Conclusion

This study established a physics-validated, data-driven framework for the rapid spatiotemporal prediction of sneeze-induced pollutant dispersion in densely populated enclosed environments. By integrating an ACT-driven CFD-Robotics Twin system, the research successfully replicated the non-linear kinematics of human sneezing, ensuring that the numerical boundary conditions were anchored in physiological reality. Experimental validation demonstrated that this integrated approach yields high fidelity in the dense momentum core, providing a rigorous benchmark for multiphase flow simulations.

Furthermore, the development of the spatial-preserving U-ConvLSTM architecture addressed the inherent limitations of traditional flattened neural networks. By maintaining the structural integrity of the pollutant field through symmetric skip-connections and convolutional long short-term memory layers, the model achieved implicit compliance with Eulerian continuity equations, as evidenced by a macroscopic mass error of only 0.31%.

The quantitative analysis of the predictive results provides critical insights into the dynamics of bio-aerosol propagation and associated infection risks. The findings indicate that the pathogenic core can expand to a maximum radius of 3.30 m within 1.2 s, significantly exceeding conventional 2.0-m social distancing guidelines. This rapid expansion, coupled with the identification of critical exposure windows driven by gravitational settling and thermal stratification, underscores the necessity of reconsidering spatial layouts and ventilation strategies in confined settings such as dormitory environments. Furthermore, the transition from iterative Navier-Stokes solvers to feed-forward tensor multiplications resulted in a three-order-of-magnitude computational acceleration, reducing inference latency to the millisecond regime.

In conclusion, the results demonstrate that embedding physical constraints and biological kinematics into deep learning frameworks enables a robust balance between computational efficiency and physical accuracy. The ability to translate abstract concentration fields into practical engineering metrics, such as dynamic safety radii and vertical exposure windows, offers a scalable tool for real-time risk assessment. This methodology provides a scientific foundation for the development of active HVAC control systems and the optimization of indoor architectural design, ultimately contributing to the creation of more resilient and health-oriented built environments.

To further mitigate the risk of catastrophic aerodynamic distortion in future iterations, the transition from pixel-based convolutional encoders to non-Euclidean representation learning is recommended. The failure of conventional flattened architectures stems from the irrevocable destruction of spatial topology when high-dimensional fluid fields are projected into 1D latent vectors. Future work should prioritize Graph Neural Networks (GNNs), which operate directly on the unstructured mesh nodes of the CFD domain, thereby inherently preserving the coordinate-dependent connectivity of the flow field without the need for skip-connections. Furthermore, the integration of Physics-Informed Neural Networks (PINNs) offers a promising paradigm shift; by embedding the Navier-Stokes and mass-continuity equations directly into the loss function as soft constraints, the surrogate model can be regularized to satisfy fundamental conservation laws even in data-sparse regimes. Such a hybrid approach, combining the temporal memory of ConvLSTM with the rigid physical scaffolding of PINNs, would provide an unparalleled level of hydrodynamic fidelity for real-time biosafety assessments.

## Data Availability

The raw data supporting the conclusions of this article will be made available by the authors, without undue reservation.
